# Nubp1 Is Required for Lung Branching Morphogenesis and Distal Progenitor Cell Survival in Mice

**DOI:** 10.1371/journal.pone.0044871

**Published:** 2012-09-17

**Authors:** Carsten Schnatwinkel, Lee Niswander

**Affiliations:** Howard Hughes Medical Institute, Department of Pediatrics, University of Colorado School of Medicine and Children’s Hospital Colorado, Aurora, Colorado, United States of America; Childrens Hospital Los Angeles, United States of America

## Abstract

The lung is a complex system in biology and medicine alike. Whereas there is a good understanding of the anatomy and histology of the embryonic and adult lung, less is known about the molecular details and the cellular pathways that ultimately orchestrate lung formation and affect its health. From a forward genetic approach to identify novel genes involved in lung formation, we identified a mutated Nubp1 gene, which leads to syndactyly, eye cataract and lung hypoplasia. In the lung, Nubp1 is expressed in progenitor cells of the distal epithelium. Nubp1(m1Nisw) mutants show increased apoptosis accompanied by a loss of the distal progenitor markers Sftpc, Sox9 and Foxp2. In addition, Nubp1 mutation disrupts localization of the polarity protein Par3 and the mitosis relevant protein Numb. Using knock-down studies in lung epithelial cells, we also demonstrate a function of Nubp1 in regulating centrosome dynamics and microtubule organization. Together, Nubp1 represents an essential protein for lung progenitor survival by coordinating vital cellular processes including cell polarity and centrosomal dynamics.

## Introduction

Murine lung development is initiated at embryonic day 9 (E9) by separation of the lung primordium from the anterior foregut endoderm [1], [2]. During the pseudoglandular stage from E10.5-16.5, the bronchiolar tree is established by branching morphogenesis [3] providing the foundation of the developing lung. Moreover, the epithelium begins to differentiate into secretory (Clara), neuroendocrine (NE), or ciliated cells. During the canalicular stage (E16.5-17.5), the undifferentiated distal epithelium continues to branch and gives rise to terminal sacs. During the terminal sac stage [E17.5-postnatal day (P) 5], the sacs increase in number and type 1 and 2 alveolar epithelial cells begin to differentiate. During the alveolar stage (P5-30), the terminal sacs develop into mature alveoli [4].

Molecularly, the pseudoglandular stage is marked by the expression of the transcription factor Nkx2.1 [4], [5] and the surfactant gene, surfactant protein-c (Sftpc) in the progenitor cells of the distal epithelium. In conjunction with other transcription factors such as Gata6, Nkx2.1 appears to control transcriptional programs of the respiratory system [2]. In the mouse lung, complete loss of either Gata6 or Nkx2-1 results in severe defects in branching morphogenesis and cell lineage differentiation [6], [7], [8]. As the distal progenitor epithelium grows out into the surrounding mesenchyme, a current model suggests that the progeny that are left behind in the proximal stalks change their pattern of gene expression and begin to differentiate into the various epithelial lineages. This model is supported by the downregulation of Sox9 [9]–[10] and Foxp1/2 expression [10], [11] in the progeny cells and the upregulation of markers characteristic of the proximal epithelium such as Sox2 [8]. Differentiation of epithelial cells in the airways proceeds in a proximal to distal direction, with the first evidence at E14.5. Ciliated cells, marked by the expression of Foxj1, are detected in the proximal epithelium in a “salt and pepper” pattern [11]. Cells that express the Clara cell marker, Scgb1a1 (CCSP, CC10) are detected around E15.5, in a similar pattern to Foxj1 [11].

The signaling pathways that dictate proximal-distal patterning and differentiation of both the lung endoderm and the mesoderm have been well described and include WNTs, SHH, BMPs and FGFs [1], [2]. Much less is known about the molecular details and the cellular pathways that are regulated by these signaling pathways. To discover genes that provide a better molecular understanding of early lung development and specification, we undertook an unbiased ENU-mutagenesis screen in mice and identified the gene Nubp1 to be required for distal progenitor survival and lung branching morphogenesis.

## Results

### The Mutation in Line3-2 Causes Lung Hypoplasia, Syndactyly, and Eye Cataracts

**Figure 1:**
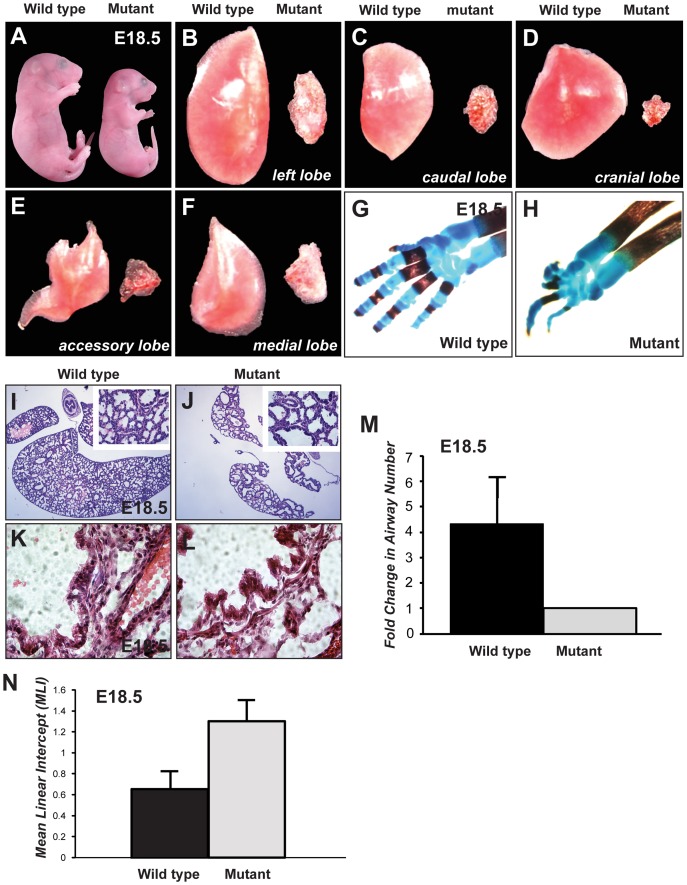
Line3-2 reveals lung hypoplasia and limb syndactyly. (A–H) Embryos dissected at E18.5 (A) reveal that line3-2 mutants are slightly reduced in size and have significantly smaller lung lobes (B–F). (G–H) E18.5 embryos fixed and stained for bone (red) and cartilage (blue) in the forelimb demonstrate gross malformation and reduction in the number of digits in the line3-2. (I–N) Histological analysis of E18.5 lung lobes show decreased airway number, increased airway diameter and defects in distal lung epithelial morphology in line3-2. (I–L) E18.5 lungs were dissected, paraffin embedded and sectioned at 10 um. (M) Airway number and (N) the mean linear intercept (MLI) of wild-type and mutant lungs were determined from 4 different embryos at E18.5. Error bars are indicated as standard error of the mean (SEM).

To identify novel genes that control lung development, we performed an ENU mutagenesis screen in mice. Embryos scored at E18.5 from line3-2 exhibited syndactyly, gross malformation and reduction in the number of digits in both hindlimbs and forelimbs (Figure 1A, G, H) and severe hypoplasia of all lung lobes (Figure 1B–F). Both, the limb and lung phenotypes were 100% penetrant and newborns did not survive due to rapid suffocation. Sections of E18.5 lungs revealed a significant decrease in airway number in homozygous mutants (Figure 1I–M), concurrent with extensive airway dilation as determined by mean linear intercept analysis (Figure 1N). The architecture of the epithelium was also disrupted. Wild type lungs showed a typical columnar epithelium, whereas the mutant epithelial cells arranged in a mushroom-like appearance and seemed to partially delaminate from the basal lamina (Figure 1K, L).

At a lower frequency (∼30%) we observed eye cataracts in homozygous mutants (data not shown). None of these phenotypes were observed in heterozygous embryos.

### Line3-2 Mutant Lungs Exhibit a Defect in Distal Cell Specification and Disruptions in Proliferation and Apoptosis

**Figure 2:**
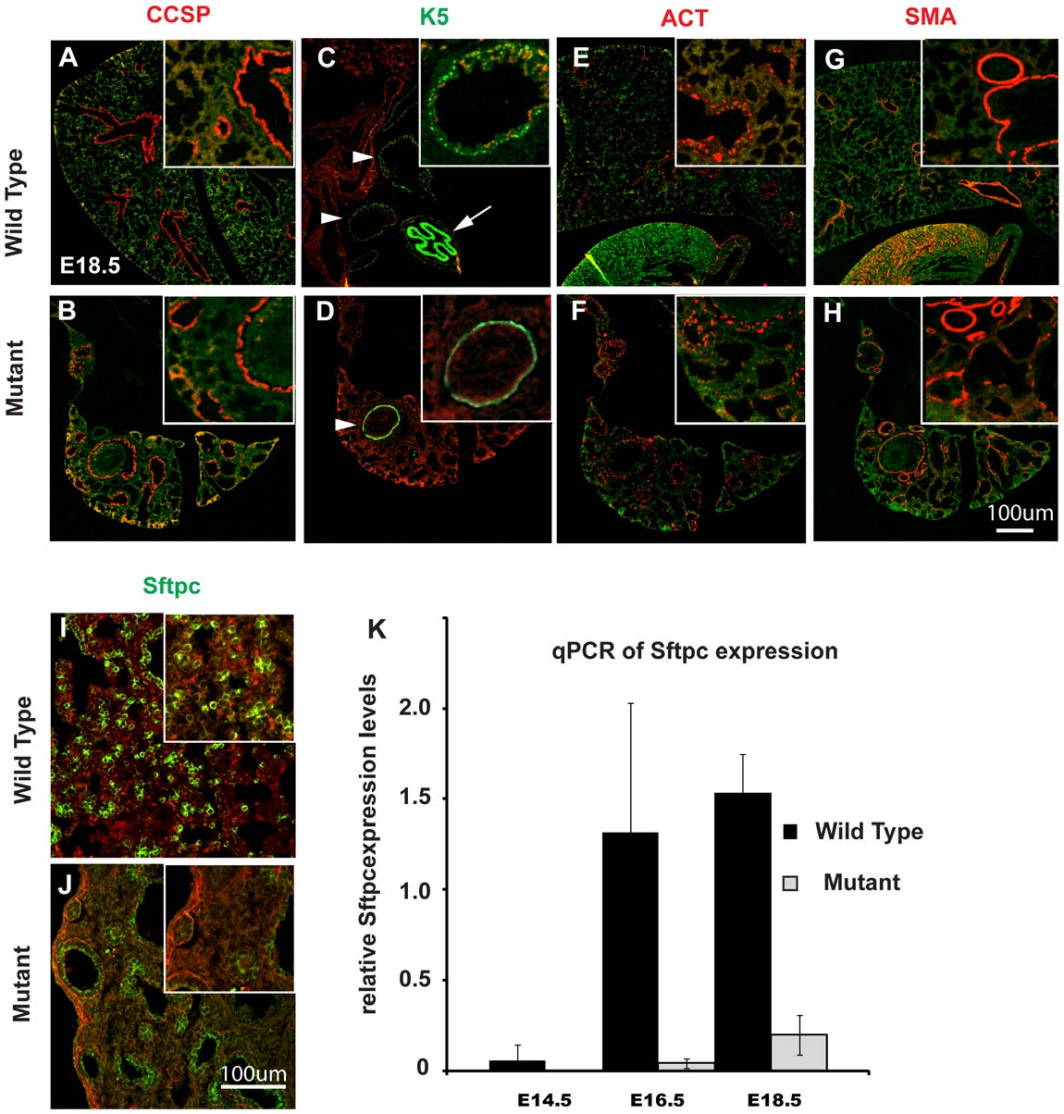
Line3-2 mutants show a reduction in distal epithelial cell marker Sftpc but normal proximal differentiation. (A–J) Lung sections from E18.5 embryos were analyzed by immunohistochemistry for the indicated proximal differentiation markers (A–H) as well as for the distal epithelial cell marker Sftpc (I–J) revealing no significant changes in proximal epithelial differentiation but a marked decrease in Sftpc expression (insets in each panel show higher magnification views). (K) Expression of Sftpc was quantified by qPCR at the indicated stages, confirming the reduced expression of Sftpc in mutant lungs. Error bars represent SEM from 4 different embryos.

Given the severe defect in branching morphogenesis, we first explored whether airway specification was disrupted. Since airway specification does not significantly start until E14.5, we decided to focus primarily on later developmental stages. In E18.5 lungs, proximal airway specification was assessed by immuno-labeling for acetylated-tubulin (all ciliated cells), CCSP (Clara cell secretory protein), and K5 (keratin-5; basal cells). The expression patterns of these markers were similar between mutant and wild type lungs, suggesting that proximal airway specification was not perturbed (Figure 2A–H). In contrast, the expression of the distal airway and progenitor marker Sftpc was significantly reduced in line3-2 mutant lungs (Figure 2I, J). The decrease in distal epithelial markers was verified by quantitative PCR (qPCR) for Sftpc, Sox9 and Foxp2 at three different embryonic stages during lung development (Figure 2K and Figure S1). Moreover, we noticed a decrease in expression levels of the transcription factor Gata6 and that the proximal epithelial marker Sox2 labeled nearly all airway epithelial cells by in situ hybridization in homozygous mutant lungs (Figure S1). These results suggest that the mutation in line3-2 is important for lung development and primarily affects the distal epithelial cell population, causing a shift towards proximal epithelial specification in the mutant lung.

**Figure 3:**
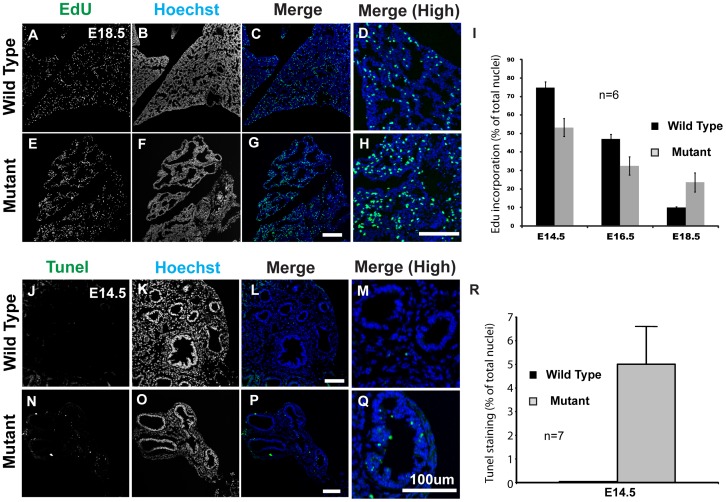
Line3-2 mutation perturbs cell proliferation and causes increased apoptosis in the lung. (A–I) In mutant lungs, cell proliferation is decreased during the pseudoglandular stage in mutant lungs, but is higher at the terminal sac stage as detected by EdU incorporation in lung sections. (I) Data presented as percentage of total nuclei. The results are representatives of 6 different sections. (J–R) Apoptosis is markedly increased in mutant lungs. (J–R) Lungs from E14.5 embryos were sectioned and processed for TUNEL staining. (R) The number of TUNEL positive cells were determined on sections and presented as percentage of total nuclei. The results are representatives of 7 different sections. Error bars are presented as SEM. (High) indicates higher magnification view.

We next investigated whether the mutation also impacts proliferation and/or apoptosis in epithelial cells. To our surprise, a short pulse of EdU at E18.5 indicated an increase in proliferating cells in mutant lungs compared to wild type (Figure 3A–H). However, at E14.5 and E16.5, proliferation was reduced ∼30% in the mutant lung compared to wild type (Figure 3I). There was also a significant increase in apoptosis as determined by TUNEL staining (Figure 3J–R). Around 5% of total nuclei in the mutant lung were TUNEL positive whereas apoptosis was barely detected in wild type at E14.5 (Figure 3R).

Taken together, our results indicate that the mutation in line3-2 affects proliferation and cell survival, as well as expression of distal cell markers. These data suggest that the severe reduction of the lung may be due to loss of distal progenitor cells leading to reduced branching and is consistent with proximalization of the developing lung.

### Line3-2 has Two Point Mutations in the Nubp1 Gene on Chromosome 16

To identify the ENU-induced mutation in line3-2 we used meiotic recombination mapping to determine association between the mutant phenotype and the C57BL/6J genetic background on which the mutation was generated. This method delimited the line3-2 mutation to a 5 Mb region between 5.3 and 11.36 Mb on chromosome 16.

**Figure 4:**
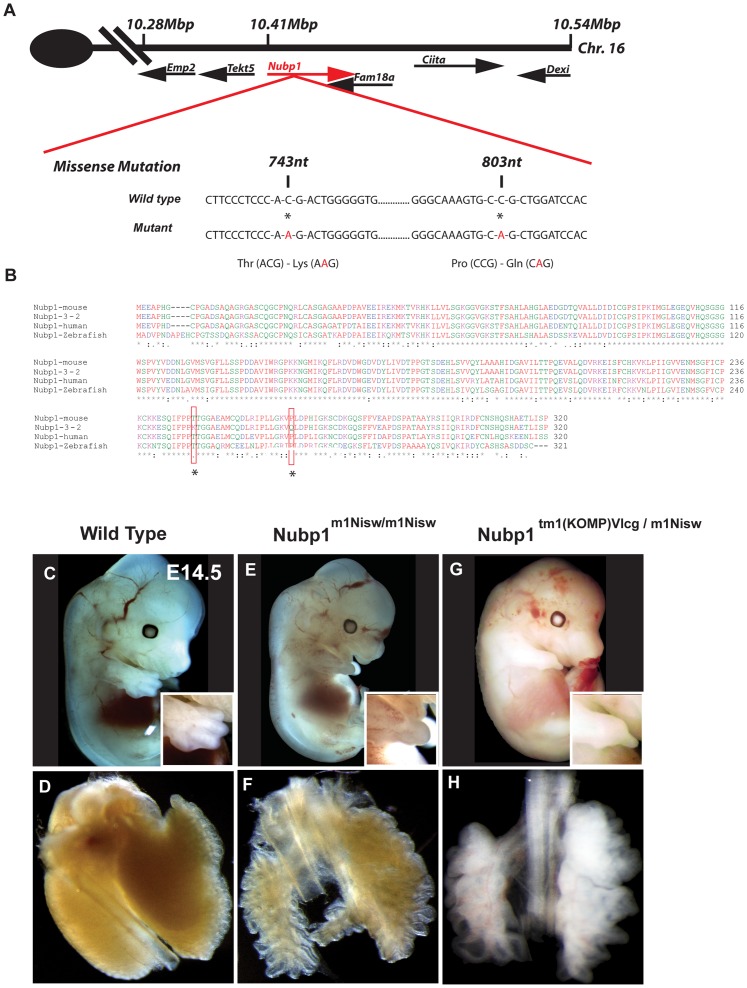
Line3-2 contains two missense mutations in the Nubp1 gene. (A) Schematic representation of the genomic region around the Nubp1 gene, which contains two missense mutations at nucleotide position 743 and 803 of the coding sequence. The mutations leads to a Threonine to Lysine and Proline to Glutamine change in the amino acid sequence of Nubp1. (B) Protein sequence alignment of the indicated genes show the mutations in line3-2 to be in a highly conserved region. Red boxes and asterisks highlight mutated regions. (C–H) E14.5 embryos from a complementation cross between line3-2 and Nubp1 knock-out reveals similar defects in lung (H) and limb development (G) as lin3-2 homozygous mutants (E, F).

Due to an infrequent recombination rate in this region (the interval was not further refined in examination of 76 meioses), the large number of genes within the interval and the potential for mutations in regulatory elements, we decided to use genomic sequence capture followed by high-throughput sequencing [12]. Comparative analysis of the lin3-2 sequencing results with C57BL/6J wild type sequences revealed two C to A transversions at position 743nt and 803nt within the coding sequence of the gene encoding nucleotide binding protein 1 (Nubp1) (Figure 4A). The two mutations were confirmed by genomic DNA sequencing using locus specific primer sets for the Nubp1 gene. The mutations resulted in a Threonine to Lysine and Proline to Glutamine change, respectively. These amino acids are highly conserved among human, mouse and zebrafish (Figure 4B) and their disruption most likely would alter protein function.

To verify that mutation of Nubp1 is responsible for the phenotypes, we obtained ES cells containing a targeted mutation in Nubp1 from EUCOMM (Nubp1^tm1(KOMP)Vlcg^, considered a definitive null allele design). After generating animals with the targeted allele, they were crossed with heterozygous line3-2 mice for a complementation assay. The resulting transheterozygous line3-2/Nubp1^tm1(KOMP)Vlcg^ embryos exhibited similar defects as line3-2 homozygous mutants (syndactyly and severely hypoplastic lungs) (Figure 4C–H). Thus, we renamed line3-2 as *Nubp1^m1Nisw^*.

### Nubp1^m1Nisw^ is Stably Expressed in the Distal Lung Epithelium and Disrupts the Interaction with Nubp2

Nubp1 and its family member Nubp2 are highly conserved proteins in eukaryotes and their homologs in Saccharomyces cerevisiae (Nbp35 and Cfd1, respectively) are essential for viability [13], [14]. Vertebrate Nubp1 and Nubp2 have a high degree of amino acid similarity (51% amino acid identity and 71.5% similarity; MATCHER) and both proteins share a nucleotide binding motif near the N-terminus. Nubp1 and Nubp2 have been reported to form a hetero-complex, suggesting that these proteins function cooperatively in vivo [15].

**Figure 5:**
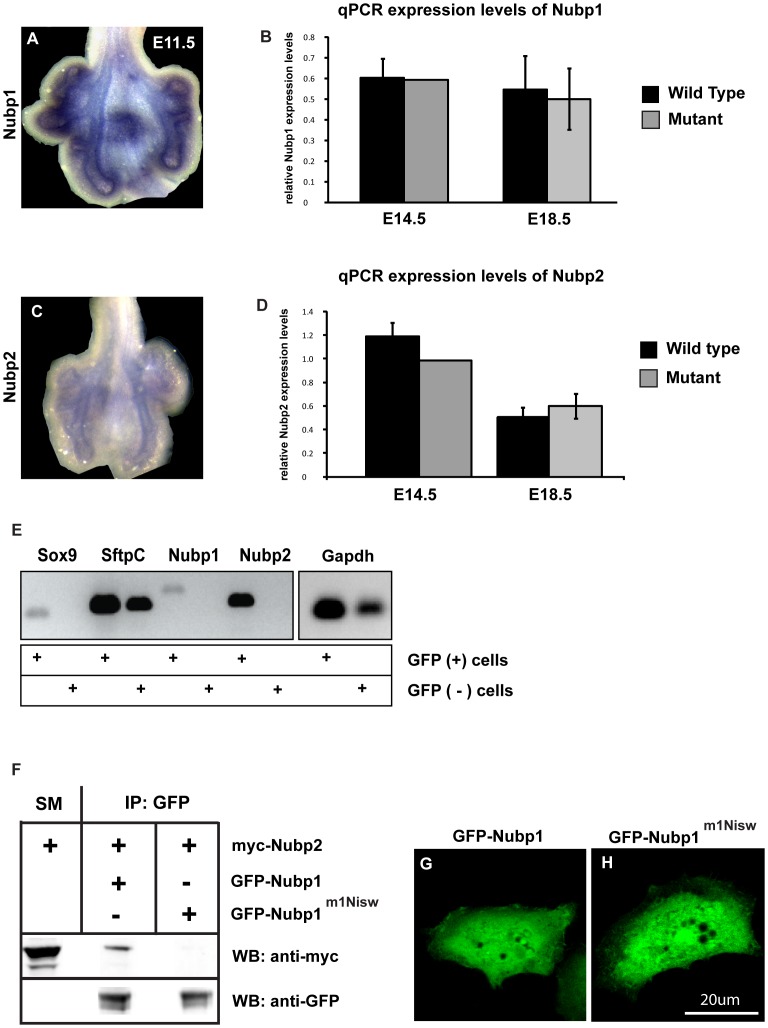
Localization, expression and interaction of Nubp1. (A–E) Nubp1 and its homolog Nubp2 are expressed in the distal lung epithelium. (A, C) In situ hybridization of Nubp1 and Nubp2 on E11.5 lung whole mounts. (B, D) Q-PCR for Nubp1 and Nubp2 at different developmental stages reveals no expression differences in mutant versus wild-type lungs. (E) PCR amplification of the indicated genes on FACS sorted E18.5 distal lung epithelial cells (GFP +) versus remaining GFP (-) cells. (F) Nubp2 interacts with Nubp1 but not with the mutant protein (Nubp1-m1Nisw). A549 cells overexpressing the indicated genes were processed for immunoprecipitation and analyzed by western blotting. (G–H) Mutation in the Nubp1 gene (Nubp1-m1Nisw) does not alter its cellular localization. A549 cells were stably transfected with GFP-Nubp1 or GFP-Nubp1(m1Nisw) and processed for immunofluorescence.

In order to test this hypothesis during lung development, we initially examined whether both genes are expressed in the same tissue. In situ hybridization for *Nubp1* and *Nubp2* showed strong expression levels for both genes in the distal lung epithelium of E11.5 wild type lungs (Figure 5A, C). We confirmed the predominantly distal expression of Nubp1 and Nubp2 by PCR using RNA from isolated distal epithelial cells and comparing it to RNA expression from the remaining cell populations in E18.5 lungs (Figure 5E).

We next determined whether the mutation in the *Nubp1* gene causes destabilization of either *Nubp1* or *Nubp2* message. To address this, we performed qPCR for *Nubp1* and *Nubp2* on wild type and *Nubp1^m1Nisw^* lungs at E14.5 and E18.5. However, we did not detect a significant change in expression of either gene in mutant compared to wild type lungs (Figure 5B, D).

To test whether mutant Nubp1 can still interact with Nubp2, we co-expressed myc-tagged Nubp2 with either GFP-tagged wild type or *Nubp1^m1Nisw^* mutant in HeLa cells, performed immunoprecipitation with anti-GFP antibody and analyzed the immunoprecipitated lysate by western blot. As shown in Figure 5F, Nubp2 was detected in the wild type Nubp1 precipitate as reported previously [16], but the mutant protein did not interact with Nubp2. However, we did not detect a difference in the cellular localization of the GFP-tagged mutant fusion protein relative to GFP-tagged wild-type Nubp1, suggesting that the mutant protein is properly localized within the cell (Figure 5G, H).

Our observations that both Nubp1 and Nubp2 are expressed in distal lung epithelium and that the Nubp1 mutation disrupts its interaction with Nubp2 raises the possibility that Nubp2 may also function in lung development.

### Nubp1 is Required for Proper Centrosome Positioning

To identify potential cellular pathways that may be disrupted in the Nubp1 mutant, we used a knock-down strategy in a lung epithelial cell culture line (A549). Previous studies have suggested that mouse Nubp1 and Nubp2 are responsible for regulating centrosome duplication in NIH 3T3 mouse fibroblasts [16]. However, the mechanism underlying Nubp1/Nubp2 function in centrosome duplication or whether these proteins act in other cellular processes remains largely unknown and has never been studied in the context of lung development.

**Figure 6:**
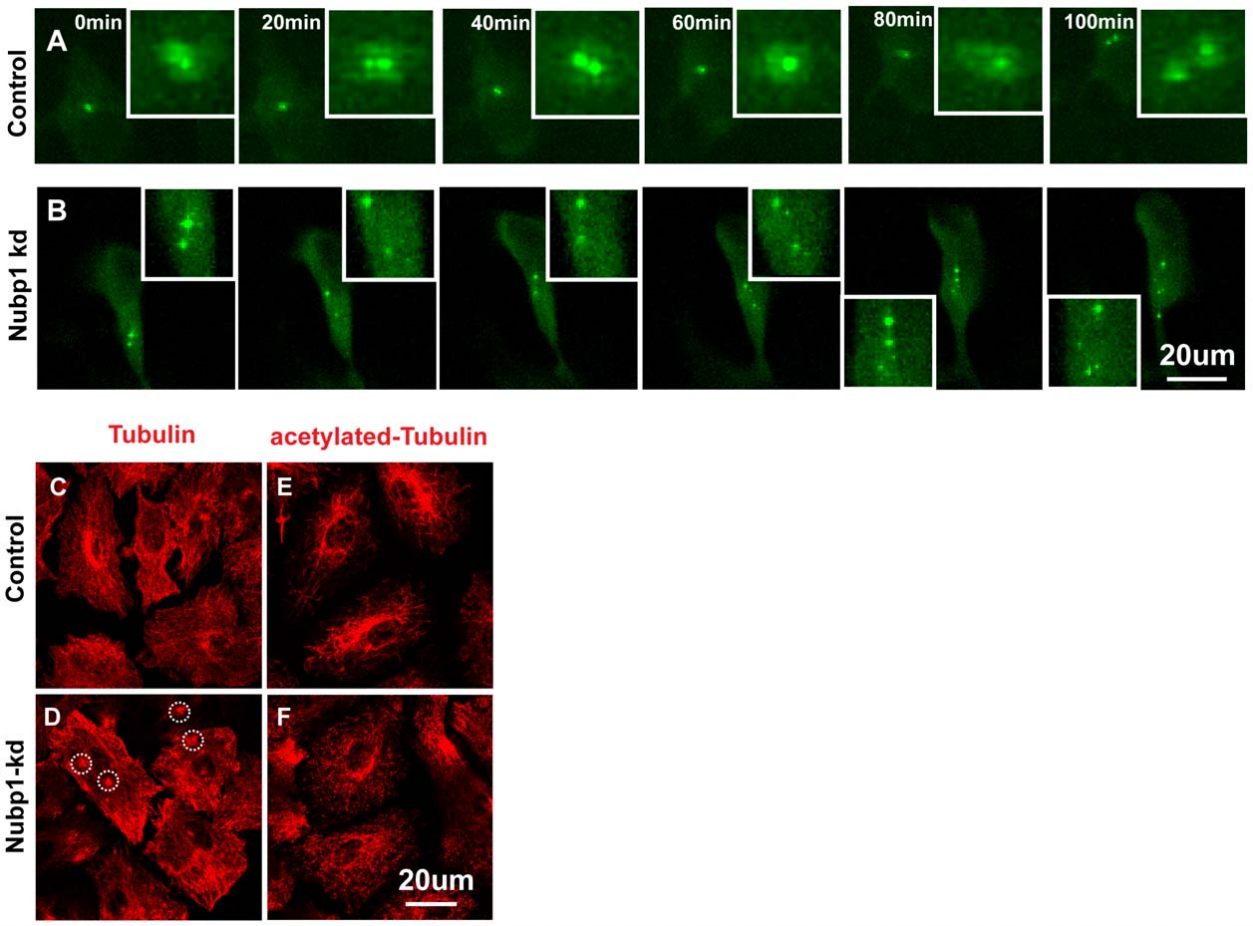
Knock-down of Nubp1 causes centrosome multiplication and alteration in centrsome dynamics. (A–B) A549 cells stably transfected with GFP-Centrin were either transfected with control or Nubp1 specific siRNA. Represented is a time sequence showing the movement of the centriole pair in control cells and the aberrant numbers, increased and irregular centrosome movements in Nubp1 knock-down cells. (C–F) Knock-down of Nubp1 leads to multiple microtubule organization points and decreased acetylated-tubulin. A549 cells were transfected with either control or Nubp1 siRNA and stained for the indicated antigens.

To visualize centrosomes in wild type and Nubp1 knock-down cells we stably expressed GFP-Centrin in A549 cells [17]. As shown before in fibroblasts, we observed multiple centrosomes in Nubp1 knock-down cells. Moreover, by visualizing centrosome dynamics over time we found that centrosome behavior was severly affected in Nubp1 knock-down cells. In control cells the GFP-tagged centriole pair stayed together during the entire image sequence (Figure 6A; Movie S1), whereas in Nubp1 knock-down cells many GFP-centrin labeled structures moved separately from each other in a seemingly random fashion (Figure 6B; Movie S2).

Since centrosomes are nucleation centers for microtubules, we sought to determine whether the changes in centrosome numbers and positioning would also alter the microtubular network. Indeed, in Nubp1 knock-down cells there were more distinct foci from where microtubules radiated (Figure 6C, D). We also stained the cells for acetylated-tubulin as an indicator of stable microtubules and this showed a marked decrease in Nubp1 knock-down cells (Figure 6E, F), suggesting that Nubp1 affects microtubule dynamics.

Whether the microtubular defects primarily occur due to centrosome perturbation or whether there are other cellular defects remains to be determined.

### Nubp1 Perturbs the Distribution of Par3 and Numb Proteins in the Developing Lung

We next sought to determine how the mutation in Nubp1 causes centrosome misregulation in the lung. Centrosome positioning is tightly coupled with the localization of the membrane associated proteins, Numb and Par3. Numb is distributed uniformly around the cell cortex in interphase cells but during mitosis Numb becomes redistributed to a small cortical region overlying one of the centrosomes [18]. Par3 associates with Dynein to regulate local microtubule dynamics and centrosome orientation [19].

**Figure 7:**
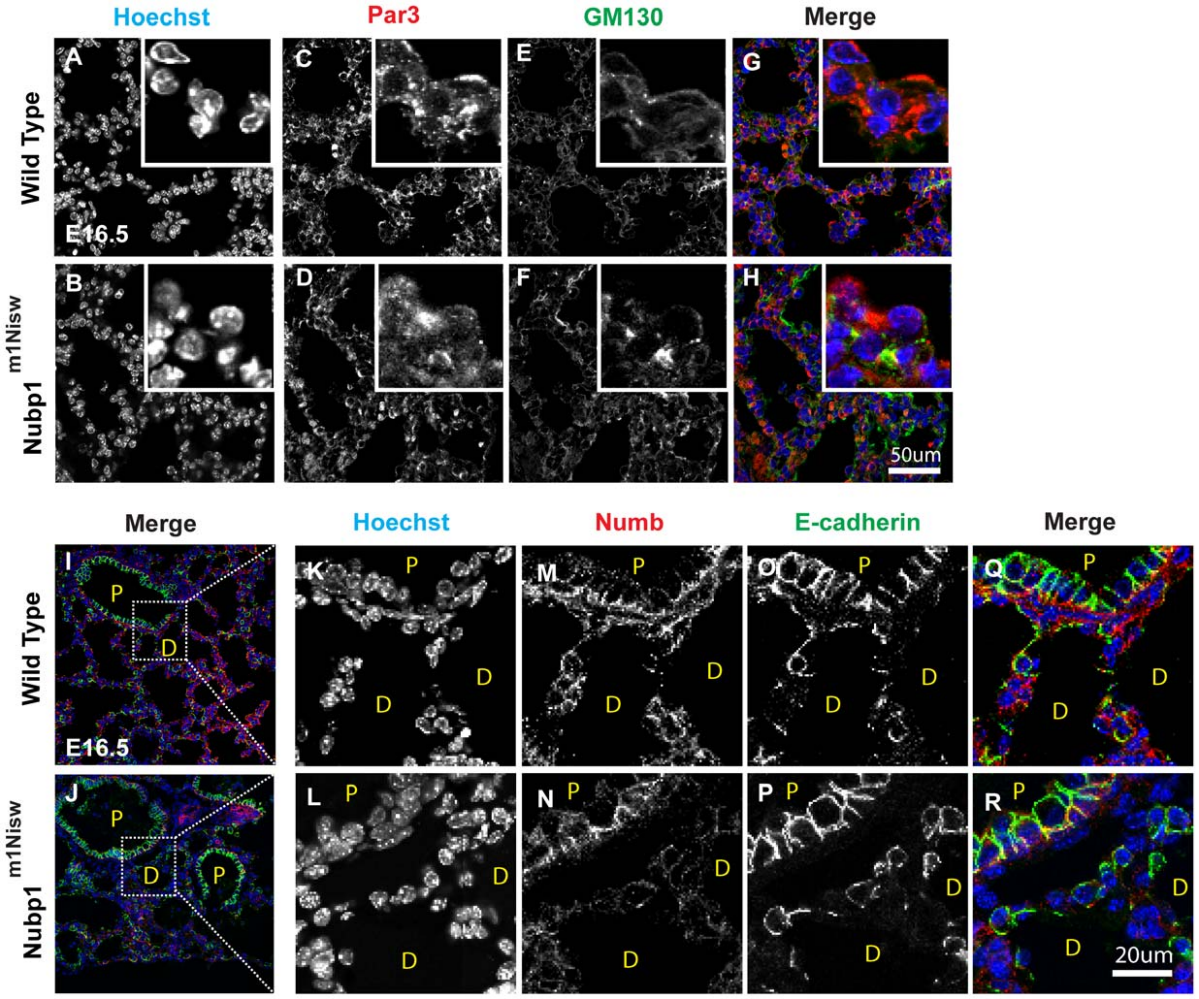
Nubp1^(m1Nisw)^ lungs show altered distribution of Par3 and Numb1. (A–R) E16.5 lungs were sectioned and stained by immunohistochemistry for the indicated proteins. (A–H) The cellular localization of the polarity protein Par3 is significantly disrupted in Nubp1^(m1Nisw)^ mutant lung. (I–R) The mutation in Nubp1^(m1Nisw)^ disrupts the cellular localization of Numb1. Note the defect is restricted to distal epithelial cells. Images K-R are higher magnifications of the regions indicated in images I and J.

To determine whether the mutation in Nubp1 affects distribution of Par3 and Numb during lung development we performed immuno-stainings on E14.5 mutant and wild-type sections. In wild type lungs, Par3 (Figure 7C) and Numb (Figure 7M) localized in a typical cortical pattern whereas in Nubp1 mutant lungs, both proteins lost this pronounced staining pattern and instead became more randomly distributed throughout the distal epithelial cells (Figure 7D, N). However, these patterns seemed to be unaffected in proximal epithelial cells in the mutant lung, consistent with our findings that Nubp1 function is primarily required in distal lung epithelial cells.

Taken together, this data suggests that Nubp1 may regulate centrosome behavior in the lung due to misregulation of Par3 and Numb at the cell cortex.

## Discussion

Lung development is governed by complex and highly coordinated processes that couple growth, patterning, and cell fate specification of the lung endoderm and mesenchyme in the formation of the tree-like branched organ. Many studies have explored the key developmental regulators that guide lung development including signaling molecules such as WNTs, BMPs, and SHH and transcription factors [1], [2]. These studies have built a robust framework within which to understand lung development. However, to fully grasp the underlying mechanisms it is necessary to also define the downstream effector genes that control the cellular processes to guide lung development. Here we have performed an unbiased forward genetic screen in mice to identify Nubp1 as an important gene for proper lung, as well as limb and eye development.

### Nubp1 in Lung Development

Nubp1 function in mammalian development had not been previously determined. During lung development, Nubp1 is expressed in the distal epithelium, which gives rise to the numerous airways. A defect in Nubp1 function, as in the *Nubp1^m1Nisw^* mouse line, interferes with the formation of distal progenitor cells as indicated by an increase in apoptosis of lung epithelial cells and a decrease in expression of the distal progenitor cell markers Sftpc, Sox9 and Foxp2. Proliferation is also significantly perturbed and the number of airways are markedly decreased. However, the differentiation of proximal epithelial cells into various cell lineages is not obviously affected.

Premature differentiation of distal progenitor cells into proximal cell fates is a possible explanation for the altered airway formation in the Nubp1 mutant, however, this would not explain the increase in apoptosis and probably would change the distribution of proximal cell markers. At E18.5 proliferation is increased in the mutant lung, suggesting that some undifferentiated cells remain proliferative, whereas the wild-type lung epithelium has ceased proliferation and switched to predominantly differentiation events.

The reduced number of distal epithelial cells likely contributes to the smaller lung size in mutant lungs, yet the airway diameter seems to be increased as indicated by the mean linear intercept analysis (MLI). Two not mutually exclusive reasons may account for this phenomena. (1) The airway diameter determined by MLI reflects predominantly the diameter of the remaining, bigger proximal airways in the mutant lung. (2) The branching defect in the mutant lungs may result in an expanded distal airway due to a defect in bifurcation and therefore contributes to the general increase in airway diameter. Our immunofluorescence analysis did not detect any significant changes in proximal airway diameter between wild type and mutant lungs, however, there was a striking loss of SftpC positive distal epithelial cells. This is consistent with the first possibility, presence of proximal airways and reduction in distal airways, as a primary reason for the apparent increase in airway diameter.

### Cellular Function of Nubp1

Our studies also begin to address the cellular function of Nubp1 in mammalian development. Knock-down studies in tissue culture cells have demonstrated that loss of Nubp1 as well as its homologue Nubp2 result in abnormal centrosome duplication [16].

Using human lung epithelial cells we see a similar defect upon Nubp1 knock-down. In addition, dynamic visualization of centrosome behavior in Nubp1 knock-down cells shows that Nubp1 regulates centrosome dynamics and positioning in the cell. Nubp1 may act directly on the centrosome or it may coordinate cellular events at the cell cortex as our data show a redistribution of the cortical proteins Numb and Par3 in Nubp1 mutants. Both Numb and Par3 play a vital role in cell division and centrosome positioning. Par3, as a member of the polarity proteins, functions in asymmetrically positioning the mitotic spindle [20], [21] and recently has been demonstrated to position centrosomes in migrating cells [19], whereas Numb1 is a key target for Par proteins during cell division [22]. In addition, the Nubp1 homologue, MinD associates with the cortical membrane to position cytoskeletal elements that dictate bacterial cell division [23].

Based on this information, Nubp1 may assist in orchestrating the proper assembly of the cortical machinery to guide centrosome positioning and cell division. Malfunction due to Nubp1 mutation could lead to abnormal behaviors such as defects in cell polarity, the number and behavior of centrosomes and ultimately to apoptosis as shown in our studies as well as previously exemplified for the yeast homologue NBP35 (Vitale, 1996, Gene).

Although, a function of Nubp1 at the level of the centrosome cannot be ruled out, cellular localization studies of Nubp1 by Okuno [24] and our lab do not reveal a predominant centrosomal or spindle localization.

### Molecular Function of Nubp1

Nubp1 is a family member of small ATPases and Nubp1 has been demonstrated to form a hetero-complex with Nubp2 [16]. Nubp1 and Nubp2 show a similar subcellular pattern of localization [24] and both genes are co-expressed in the distal lung epithelium suggesting that these proteins act together. The *Nubp1^m1Nisw^* mutation does not affect Nubp1 expression and distribution in cells and lung tissue but it does disrupt the interaction with Nubp2. Whether a disruption in this interaction is the primary consequence of the Nubp1 mutation can only be suggested and remains to be directly addressed in the future by comparing the Nubp1 mutant phenotype to that of a Nubp2 mutation. Based on previous studies which showed similar defects in Nubp1 or Nubp2 knock-down cells [16], it seems possible that the activity of both proteins are required for full functionality.

Many ATPases have multiple binding partners and therefore it seems unlikely that Nubp2 is the only interaction that Nubp1 mediates. Instead we propose a model where Nubp1 acts as a molecular switch to maintain cell polarity and proper centrosomal behavior. Both cellular processes seem to be essential for proper lung development as shown by recent studies on cell division orientation and asymmetric cell division during lung branching morphogenesis although the molecular outcome of this cellular function remains poorly understood [25], [26]). The Nubp1 mutant described here provides another powerful tool to shed new insights into aforementioned processes and to dissect the interconnections between cellular behaviors and lung morphogenesis.

## Materials and Methods

### Mouse Strains, Ethics Statement, Genotyping, and Nubp1 Mutation Identification

All mice were bred and kept in strict accordance with the recommendations in the Guide for the Care and Use of Laboratory Animals of the National Institutes of Health. Steps were taken to minimize suffering. The research protocol was approved by the Institutional Animal Care and Use Committee of the University of Colorado at Denver. The mutation in Nubp1 was identified in a screen for recessive ENU-induced mutations that cause morphological changes in lung development in E18.5 embryos [27], [28]. Mutations were induced with N-ethyl-N-nitrosourea on a C57BL/6J genetic background and outcrossed to 129S1/SvImJ (129). Line3-2 was outcrossed for more than ten generations to establish a congenic line and no phenotypic variation was observed on the different backgrounds. The line3-2 mutation was initially mapped between Massachusetts Institute of Technology (MIT) SSLP markers D16Mit33 and D16Mit56. The mutation was ultimately identified by genomic sequence capture technology [12] using Roche-Nimblegen custom designed hybrid capture microarray chip designed against unique sequence within the 5 Mb interval between 5.3 and 11.36 Mb on chromosome 16. The two mutations within the Nubp1 gene were verified by sequencing Nubp1 cDNAs generated by RT-PCR (SuperScript One-Step RT-PCR; Invitrogen) using RNA from 6 different E14.5 mutant embryos compared to wildtype control cDNA from E14.5 embryos of C57BL/6J background. A Nubp1 mouse embryonic stem (ES) cell line (JM8.F6) with a targeted mutation in Nubp1 (tm1(KOMP)Vlcg) was obtained from KOMP Repository at the University of California Davis (CloneID EPD0089_1_C03)and was used to generate Nubp1 knock-out mice by blastocyst injection. Genotyping was performed by PCR with lacZ primers [29].

### Skeletal Staining, Histology, RNA and Protein Localization

Alcian-blue and alizarin-red staining of cartilage and bone were performed as described [30]. Whole-mount and section RNA in situ hybridization were performed as described [31], [32]. Immunohistochemistry on cryosections was performed as described [33]. Antibodies: CCSP (gift from S. Reynolds; dilution 1∶5000), Keratin-5 (gift from S. Reynolds; dilution 1∶500), acetylated-Tubulin (Sigma; dilution 1∶200), human pro-surfactant protein-c (Seven Hills, dilution 1∶200), Par3 (Millipore, dilution 1∶100), GM130 (BD Biosciences, dilution 1∶200), Numb (Cell Signaling, dilution 1∶200), Smooth muscle actin (Sigma, dilution 1∶500), Cy5 labeled beta-tubulin (Sigma, dilution 1∶500), Hoechst (Molecular probes, dilution 1∶500), E-cadherin (Transduction Laboratories; dilution 1∶500). Immunofluorescence images were taken on a LSM510 META confocal microscope from Zeiss. EDU (1 mg/20 g body weight) was injected intraperitoneally into pregnant females 1 hr before sacrifice and detected according to the manufacturer’s procedure (Invitrogen).?EDU incorporation and TUNEL staining for apoptotic cells, were quantified in sections from the indicated stages of wild-type or Nubp1 mutants, comparing the number of positive nuclei to the total number of nuclei in the field. Mean linear intercept was determined as described previously [34]. To determine the number of airways, sections of mutant and wild-type lungs were obtained and the number of airways in representative areas were counted.

### FACS Sorting and PCR Analysis

Transgenic mice in which epithelial GFP was expressed under the human Sftpc-promoter were harvested at E18.5, mildly homogenized in BGjB medium to obtain single cell suspension and FACS sorted for GFP expressing (+) versus non-expressing cells (-). RNA was extracted using Phenol/Chloroform and transcribed into DNA using SuperScript One-Step RT-PCR from Invitrogen. 50 ng of DNA were used for PCR analysis using the following primers: Nubp1 forward primer: CCCCAACCAGAGGCTGTGCG, reverse primer: CCGGACATCCTGGAGGGCCA; Nubp2 forward primer: CTGCCGGTGAGCGTGCAGAA, reverse primer: AGGGGCGCAGGGCTTCCATA; Sox9 forward primer: CCCTTCGTGGAGGAGGCGGA, reverse primer: CGTCGCGGAAGTCGATGGGG; Sftpc forward primer: TCCCAGGAGCCAGTTCCGCA, reverse primer: TGCCCTTCCTCCTGGCCCAG; Gapdh forward primer: CTGGCACTGCACAAGAAGATGC, reverse primer: GGGTTCCTATAAATACGGACTGCAGC.

### Cloning, Coimmunoprecipitation and Cell Cultures

Full length cDNAs for Nubp1, Nubp2 and Nubp1^m1Nisw^ were obtained by PCR from wild-type 129S1/SvImJ mice or *Nubp1^m1Nisw^* mutant embryos using the following primers. Nubp1 (wt and mutant), forward primer: 5?-actgagaattcatggaggaggcgccccac-3¢, reverse primer: 5¢-tgtgtctcgagtcagggactgatgagggtctcag-3¢. Nubp2, forward primer: 5?- actgactcgag**atggaggctgctgccggtg**-3¢, reverse primer: 5¢- tgtgtctcgagtcaggagcacagggcagaca -3¢. cDNAs were subcloned into pEGFP and pCMV3-myc expression vectors. Hela cells or A549 cells were obtained from ATCC and transfected with the indicated plasmids using Lipofectamine 2000 Plus (Invitrogen). For immunoprecipitation, cell lysates were incubated with 1 ug of anti-GFP antibody (Sigma, 1∶1000 dilution) and western blotting was performed with anti-myc or anti-Nubp2 antibody (Sigma, 1∶1000 dilution). For life cell imaging, A549 cells were transfected with a pEGFP-Centrin plasmid (kindly provided by Dr. Bornens). Stable clones were selected using G418 (4 ug/ml). Nubp1 knock-down was generated by RNAi using 4 different siRNA targets for Nubp1 which were transfected with RNAifect (Qiagen).

### Time-Lapse Microscopy

Confocal time-lapse movies were collected on a Zeiss 7 LIVE microscope by using a C-Apochromat 40x/1.2 W Korr objective lens. Temperature was held at 37°C and CO_2_ was held at 5% by using a CTI Controller 3700 and Temperature Control 37.2 combination. Images were acquired every 2 min. Images collected were analyzed using Imaris (Bitplane) and Zen software (Zeiss). Representative single experiments are shown.

## Supporting Information

Figure S1 **Line3-2 reveals a decrease in distal lung epithelial markers.** (A–F) Sections from lungs of E16.5 wild type and mutant embryos were prepared for in situ hybridization using the indicated genes. (G–I) Q-PCR was performed on wild type and mutant embryos for the indicated embryonic stages. Note the decrease of the distal lung epithelial markers Sox9 and Foxp2 by q-PCR and the labeling of all airways with the proximal marker Sox2 by in situ hybridization in mutant lungs. The results are representatives from at least 4 different embryos. Error bars are presented as SEM.
(TIF)

Movie S1 **Confocal Time-Lapse Movie of GFP-Centrin stably expressing A549 cells and transiently transfected with control siRNA.** A549 cells stably expressing GFP-Centrin were transiently transfected with 50 nM non-silencing control siRNA for 3 days. Images were taken every 2 min. Movie plays at 7fps. Still images from this movie are shown in Figure 6A.
(MOV)

Movie S2 **Confocal Time-Lapse Movie of GFP-Centrin stably expressing A549 cells and transiently transfected with Nubp1 specific siRNA.** A549 cells stably expressing GFP-Centrin were transiently transfected with 50 nM of Nubp1 specific siRNA for 3 days. Images were taken every 2 min. Movie plays at 7fps. Still images from this movie are shown in Figure 6B.
(MOV)
