# Supplementary figures and images for: Nubp1 Is Required for Lung Branching Morphogenesis and Distal Progenitor Cell Survival in Mice
Source: PLoS One. 2012 Sep 17;7(9):e44871. doi: 10.1371/journal.pone.0044871 (PMC3444492; doi:10.1371/journal.pone.0044871)

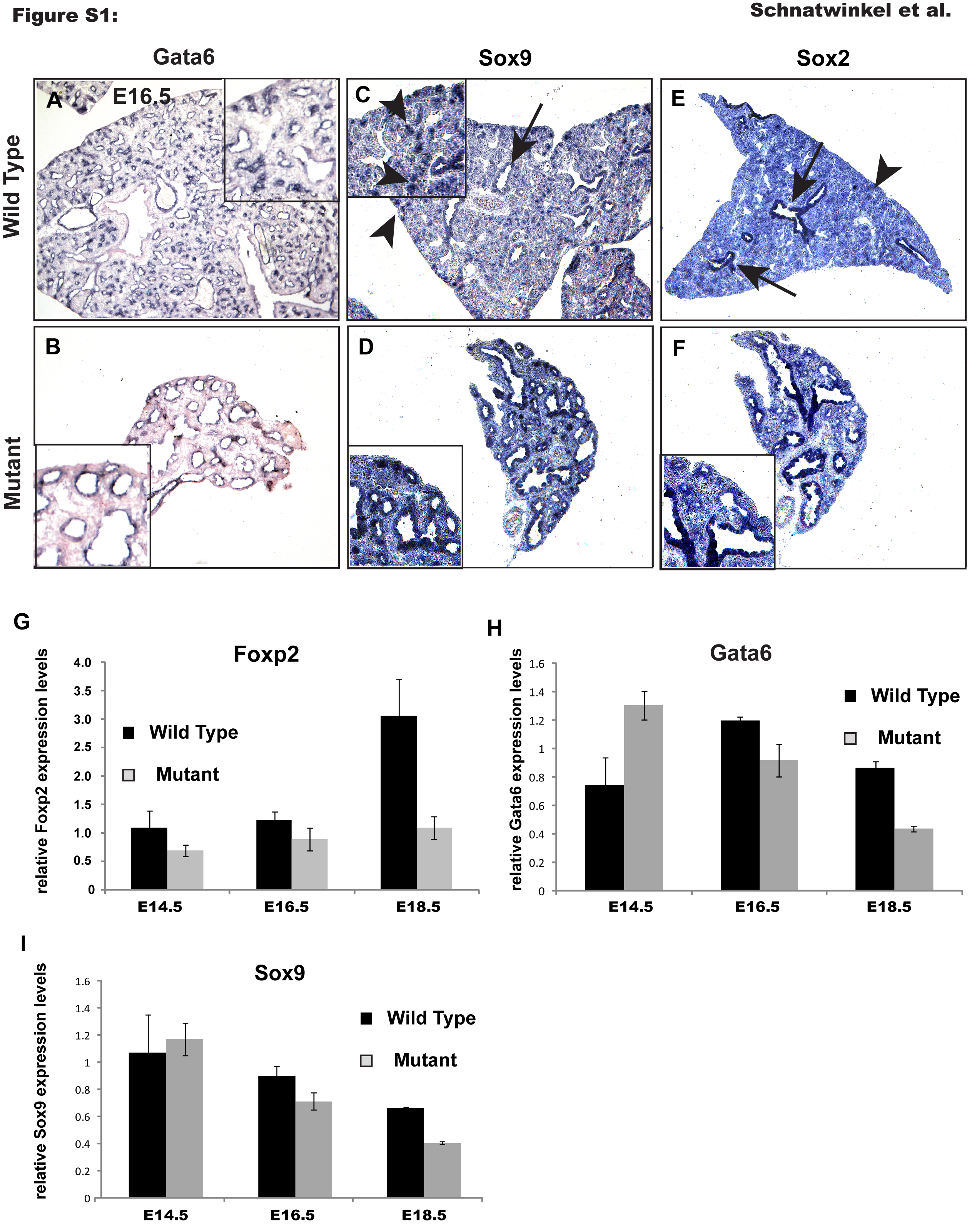

Supplement: Figure S1 — Line3-2 reveals a decrease in distal lung epithelial markers. (A–F) Sections from lungs of E16.5 wild type and mutant embryos were prepared for in situ hybridization using the indicated genes. (G–I) Q-PCR was performed on wild type and mutant embryos for the indicated embryonic stages. Note the decrease of the distal lung epithelial markers Sox9 and Foxp2 by q-PCR and the labeling of all airways with the proximal marker Sox2 by in situ hybridization in mutant lungs. The results are representatives from at least 4 different embryos. Error bars are presented as SEM. (TIF) [file pone.0044871.s001.tif]
